# Correlation between the BACTEC MGIT 960 culture system with Genotype MTBDR*plus* and TB-SPRINT in multidrug resistant *Mycobacterium tuberculosis* clinical isolates from Brazil

**DOI:** 10.1590/0074-02760170062

**Published:** 2017-11

**Authors:** Nayanne Gama Teixeira Dantas, Phillip Noel Suffys, Wânia da Silva Carvalho, Harrison Magdinier Gomes, Isabela Neves de Almeida, Lida Jouca de Assis Figueiredo, Alan Douglas Gonçalves, Michel Kireopori Gomgnimbou, Guislaine Refregier, Christophe Sola, Silvana Spíndola de Miranda

**Affiliations:** 1 Universidade Federal de Minas Gerais, Faculdade de Medicina, Departamento de Clínica Médica, Programa de Pós-Graduação em Infectologia e Medicina Tropical, Belo Horizonte, MG, Brasil; 2 Fundação Oswaldo Cruz-Fiocruz, Instituto Oswaldo Cruz, Laboratório de Biologia Molecular Aplicada a Micobactéria, Rio de Janeiro, RJ, Brasil; 3 Universidade Federal de Minas Gerais, Faculdade de Farmácia, Departamento de Farmácia Social, Laboratório de Biologia Molecular e Saúde Pública, Belo Horizonte, MG, Brasil; 4 Fundação Ezequiel Dias, Belo Horizonte, MG, Brasil; 5 Centre Muraz, Bobo-Dioulasso, Burkina Faso; 6 Institut for Integrative Cell Biology, UMR9198 CEA-CNRS-UPSaclay, Orsay, France; 7 University Paris-Sud, Beamedex SAS, Orsay, France

**Keywords:** tuberculosis, multidrug-resistant, genotype, molecular diagnosis

## Abstract

**BACKGROUND:**

The accurate detection of multidrug-resistant tuberculosis (MDR-TB) is critical for the application of appropriate patient treatment and prevention of transmission of drug-resistant *Mycobacterium tuberculosis* isolates. The goal of this study was to evaluate the correlation between phenotypic and molecular techniques for drug-resistant tuberculosis diagnostics. Molecular techniques used were the line probe assay genotype MTBDR*plus* and the recently described tuberculosis-spoligo-rifampin-isoniazid typing (TB-SPRINT) bead-based assay. Conventional drug susceptibility testing (DST) was done on a BACTEC^TM^ MGIT 960 TB.

**METHOD:**

We studied 80 *M. tuberculosis* complex (MTC) clinical isolates from Minas Gerais state, of which conventional DST had classified 60 isolates as MDR and 20 as drug susceptible.

**FINDINGS:**

Among the 60 MDR-TB isolates with MGIT as a reference, sensitivity, specificity, accuracy, and kappa for rifampicin (RIF) resistance using TB-SPRINT and MTBDR*plus*, were 96.7% versus 93.3%, 100.0% versus 100.0%, 97.5% versus 95.0% and 0.94 versus 0.88, respectively. Similarly, the sensitivity, specificity, accuracy, and kappa for isoniazid (INH) resistance were 85.0% and 83.3%, 100.0% and 100.0%, 88.8% and 87.5% and 0.74 and 0.71 for both tests, respectively. Finally, the sensitivity, specificity, accuracy, and kappa for MDR-TB were 85.0% and 83.3%, 100.0% and 100.0%, 88.8% and 87.5% and 0.74 and 0.71 for both tests, respectively.

**MAIN CONCLUSIONS:**

Both methods exhibited a good correlation with the conventional DST. We suggest estimating the cost-effectiveness of MTBDR*plus* and TB-SPRINT in Brazil.

Tuberculosis (TB) remains an important global health problem and is responsible for the second-highest mortality rate worldwide due to a single infectious agent, the *Mycobacterium tuberculosis* complex (MTC). The World Health Organization (WHO) estimates that in 2015, 10.4 million new TB cases and 1.4 million deaths occurred. Among these incident cases, approximately 3.9% of new cases and 21% of previously treated cases were multidrug-resistant (MDR) TB, defined as resistance to at least rifampicin (RIF) and isoniazid (INH). In the same period, among the 6.1 million new and previously treated TB patients, an estimated 580,000 cases of multidrug-resistant MDR-TB could have been detected if proper drug resistance testing had been performed; however only 20% of these cases, i.e., 125,000, were detected and reported (WHO 2016).

Brazil is included in the list of the 20 countries that account for 84% of all global cases of TB. 84,000 new cases were estimated in 2015 with an incidence of 41 per 100,000 and a mortality rate of 2.7 per 100,000 (WHO 2016). Despite Brazil not being among the high-burden MDR-TB countries (WHO 2016), the number of MDR-TB cases in Brazil identified after 2007 almost doubled (from approximately 300 cases in 2007 to 702 cases in 2014) (Bollela et al. 2016). In the state of Minas Gerais, the second most populated state in the southeast region of the country, the mean MDR TB rate was 0.2% among the notified TB cases between 2002 and 2009 (Augusto et al. 2013). The last epidemiological report of Minas Gerais state described 14 new MDR TB cases in the first semester of 2013 (MS/SVS 2014).

The emergence of MDR-TB is a major issue in the control of TB because treatment becomes more difficult and costly, with increased failure rates and more adverse effects. Crucial elements of MDR-TB control include quick and cost-efficient identification of MDR-TB cases, classification of resistance type and fast implementation of the appropriate treatment scheme to interrupt transmission (Campbell et al. 2011). Laboratory confirmation by culture and identification of MTC is the best approach to guarantee proper diagnosis of any suspected cases of TB and MDR-TB (WHO 2016). However, the efficiency of culture-based drug susceptibility testing (DST) is affected by the slow growth characteristic of MTC, which can take two to eight weeks. The use of molecular methods to identify mutations associated with drug resistance can decrease the diagnostic delay and may provide accurate and rapid predictive drug susceptibility results. Continuous research in this area has promoted the development of numerous commercial and laboratory-designed diagnostic assays (Campbell et al. 2011).

Molecular-based methods for the detection of resistance in MTC have been developed based on the knowledge of genetic mechanisms causing drug resistance. An increasing number of genes are related to phenotypic drug resistance. Mutations within an 81-bp rifampin resistance determining region (RRDR) of the *rpoB* gene (codons 507-533) cause RIF resistance in approximately 95% of all clinical isolates examined, and the presence of *rpoB* mutations detected by a genotypic test is almost always associated with resistance (Drobniewski et al. 2007). In contrast, the INH drug resistance has been associated with mutations in at least four genes, with the main genetic regions involved being the *katG* gene and the *inhA* gene promoter (Folkvardsen et al. 2013).

In the last 15 years, genotypic methods have been developed for the detection of chromosomal mutations that confer drug resistance in MTC. Among these, the INNOLiPA (Innogenetics, Ghent, Belgium) and the line probe assay (LPA) GenoType MTBDR*plus* (Hain Lifescience GmbH, Nehren, Germany) have been recommended by the WHO for “In Vitro Diagnostics” (WHO 2008). Recently, Tuberculosis-spoligo-rifampin-isoniazid typing (TB-SPRINT; Beamedex, Orsay, France) was launched as a “Research Use only” test. This assay detects resistance to first-line drugs by analysis of the *rpoB, katG* and *inhA* genes and simultaneously determines the spoligotyping pattern that may give some clues about MDR-TB transmission cases, as such helping to optimise personalised medicine and the public health issue related to prevalence of particular lineages and recent transmission. Both methods are based on polymerase chain reaction (PCR) amplification of genomic regions where mutations associated with resistance are common and detect either a set of specific mutations positions (e.g., positions 516, 526, 531 in *rpoB*) by hybridisation with specific probes or unspecified mutations by lack of signal from wild-type (WT) directed probes (Gomgnimbou et al. 2013).

In June 2008, the WHO recommended the use of MTBDR*plus* for detection of MDR-TB (WHO 2008) and in December 2010, endorsed the Xpert MTB/RIF (Cepheid, USA) technology, based on the GeneXpert System, as promising because it is used directly on clinical specimens, such as sputa, avoiding delay due to specimen treatment and DNA extraction and providing the so called “sample-to-answer” solution. However, the MTBDR*plus* and Xpert MTB/RIF require expensive equipment and/or reagents: the cost of Xpert testing is around US $15 and similar to that of a combination of smear-microscopy followed by liquid culture and conventional DST (about US $17) while the MTBDR*plus* assay is even more costly (around US $24) (Shah et al. 2013). The TB-SPRINT assay can detect the *rpoB* gene RRDR mutation with a sensitivity (90.4%) and specificity (100%) for MDR detection similar to MTBDR*plus* or GeneXpert MTB/Rif, with a lower cost (around US $10) and a higher throughput, allowing a screening of isolates before eventual sequencing (Gomgnimbou et al. 2013).

Unlike the other genotype-based assays, TB-SPRINT has been evaluated in a limited number of studies to date. Moreover, there were no previous studies demonstrating its performance for MDR detection compared to MTBDR*plus*. We therefore evaluated the performance of MTBDR*plus* and TB-SPRINT in a set of *M. tuberculosis* clinical isolates from Minas Gerais state using the phenotypic drug susceptibility test (DST) BACTEC MGIT 960 culture system (Becton Dickinson Microbiology System, Sparks, NV, USA) as a reference.

## MATERIALS AND METHODS

### Study settings and population

The study is based on a convenience sample including 80 MTC isolates. Each isolate, corresponding to a unique TB patient, was collected randomly from the MTC clinical isolates collection of the Micobacteria Research Laboratory of the Faculty of Medicine of the Federal University of Minas Gerais, from 2007 to 2013, in the population of Minas Gerais state. Their phenotypic profiles were 20 drug- susceptible and 60 MDR.

### Conventional drug susceptibility testing

Clinical isolates from distinct local laboratories in Minas Gerais state were referred to the Ezequiel Dias Foundation (FUNED) to perform culture, identification and DST. This TB reference centre has External Quality Assurance. The culture-based DST used was the BACTEC^TM^ MGIT 960 System method. Drug susceptibility kits were available for testing of INH (0.1 μg/mL) and RIF (1.0 μg/mL).

### MTBDRplus

The GenoType^®^ MTBDR*plus* version 2.0 assay was performed for all strains according to manufacturer's instructions, as described previously (Aurin et al. 2014).

### TB-SPRINT

High-throughput TB-SPRINT was performed and analysed on all clinical isolates on a Luminex^TM^ 200 flow cytometry device in standard 96-well SBS plates (Luminex Corp, Austin, TX) using a microbead-based DNA array method as described previously (Gomgnimbou et al. 2013).

### Sequencing

Sequencing of the RDR of the *rpoB* (codon 507-533), *katG* (codon 315) and promoter region of *inhA* was performed for the isolates with discordant results for rifampicin and isoniazid according to conventional DST and between both molecular methods (GenoType MTBDR*plus* and TB-SPRINT), as described previously (de Oliveira et al. 2003) using an ABI Prism 3100xl DNA sequencer (Applied Biosystems). Nucleotide sequences were analysed by the Applied Biosystems DNA Sequencing Analysis Software v5.2.

### Statistical analysis

Statistical analysis was performed for definition of sensitivity, specificity, accuracy and kappa statistics. This was based on the proportion of RIF, INH and MDR mutations identified by MTBDR*plus* and TB-SPRINT in MDR and susceptible clinical isolates, compared to the culture-based DST, which was used as the standard method. Specificity was defined as the proportion of true WT profiles (identified by culture-based DST) among predicted WT profiles (as identified by the molecular method assessed). All data were computed in an Excel spreadsheet (Braile & Godoy 1999).

### Ethics

The study was approved by the Ethics Committee of Federal University of Minas Gerais (No. 122.941).

## RESULTS

Conventional DST identified 60 isolates as being MDR and 20 isolates as being drug susceptible. The results obtained with both genotyping procedures correlated to those of the BACTEC^TM^ MGIT 960 System method as shown in Table I.

**TABLE I:** Patterns of drug susceptible and multi-drug resistant *Mycobacterium tuberculosis* isolates using MTBDR*plus* and tuberculosis-spoligo-rifampin-isoniazid typing (TB-SPRINT)

	MTBDR *plus*				TB-SPRINT			
Gene	Band	Gene region/mutation	Susceptible isolates (n = 20)	MDR isolates (n = 60)	Probe	Gene region/mutation	Susceptible isolates (n = 20)	MDR isolates (n = 60)
rpoB	WT1	506-509	20	60	Spa_WT1	509-514	20	57
	WT2	510-513	20	58				
	WT3	513-517	20	50	WT516	516	20	51
	WT4	516-519	20	52	Spa_WT2	517-522	20	58
	WT5	518-522	20	58				
	WT6	521-525	20	59				
	WT7	526-529	20	35	WT526	526	20	36
	WT8	530-533	20	38	WT531	531	20	36
	MUT1	Asp516Val	0	7	MUT1	Asp516Val	0	5
	MUT2A	His526Tyr	0	6	MUT2A	His526Tyr	0	5
	MUT2B	His526Asp	0	9	MUT2B	His526Asp	0	12
	MUT3	Ser531Leu	0	20	MUT3A	Ser531Leu	0	19
					MUT3B	Ser5*31*Trp	0	2
katG	WT	315	20	13	WT	315	20	13
	MUT1	Ser315Thr(1)	0	42	MUT1	Ser315Thr	0	43
	MUT2	Ser315Thr(2)	0	1	MUT2	Ser315Asn	0	4
inhA	WT1	-15/-16	20	45	WT1	-15	20	45
	WT2	-8	20	56				
	MUT1	C15T	0	14	MUT1	C15T	0	15
	MUT2	A16G	0	0				
	MUT3A	T8C	0	0				
	MUT3B	T8A	0	0	MUT2	T8A	0	0

MDR: multidrug resistant tuberculosis; WT: wild-type.

### MTBDRplus

Among the 80 clinical isolates, the MTBDR*plus* identified 24 isolates as being drug susceptible, six as RIF resistant and 50 as MDR. Fifty-six (93%) isolates were typed at the RRDR locus as a mutant genotype, 42 of which (75% of total) carried well-known mutations conferring RIF resistance. Among the RIF mutations, 20 (36%) were found at *rpoB* 531 codon (Ser531Leu mutation), 16 (29%) at codon 526 [nine His526Asp mutations (16%) and seven His526Tyr mutations (13%)], and six (11%) at codon 516 with the Asp516Val mutation. Among the isolates known to be RIF-resistant by the phenotypic DST (n = 60), 56 of them carried a mutation according to MTBDR*plus*.

When assessing INH resistance among the 80 clinical isolates by MTBDR*plus*, 50 had a mutant genotype either in the *katG* gene or in the *inhA* promoter. Among these, 47 (94%) carried well-known mutations that conferred INH resistance: 32 *katG* were Ser315Thr1 (64%), one *katG* was Ser315Thr2 (2%), four *inhA* were C15T (8%) and 10 (20%) carried mutations in both regions (*katG* Ser315Thr1 and *inhA* C15T). The mutations of the remaining INH resistant isolates could not be specified by the present assay. Among INH resistant isolates by the phenotypic DST (n = 60), 50 of them carried the INH mutation according to MTBDR*plus*.

### TB-SPRINT

Among the 80 clinical isolates the TB-SPRINT method identified, 22 were drug susceptible, seven were RIF resistant and 51 were MDR. Fifty-eight isolates typed at the RRDR locus had a mutant genotype, 44 of which (76% of total) carried well-known mutations conferring RIF resistance. Among the RIF mutations, 21 (37%) were found at the *rpoB* gene in codon 531 [19 were Ser531Leu (33%) and two were Ser531Trp (3%)], 17 (29%) at codon 526 [12 were His526Asp (21%) and five were His526Tyr (8%)], and six at codon 516 in Asp516Val (10%). Among the isolates known to be RIF resistant by the phenotypic DST (n = 60), 58 of them carried a mutation according to TB-SPRINT.

Regarding the results of TB-SPRINT on loci involved in INH resistance, 51 had a mutant genotype at *katG* 315 or at the *inhA* promoter, and all of them carried well-known mutations that confer INH resistance. Among these, 33 were *katG* Ser315Thr (65%), three were *katG* Ser315Asn (6%), four were *inhA* C15T (8%) and 11 isolates (21%) carried a mutation in both regions: one was *katG* Ser315Asn + *inhA*C15T (2%) and 10 were *katG* Ser315Thr + *inhA* C15T (19%).

### Sequencing

Sequencing the RRDR region of the genes *rpoB, katG* and the *inhA* promoter region of the five clinical isolates, with discordant results for RIF and INH, identified two as being drug susceptible, two as RIF resistant and one as MDR. Among these, sequencing the RRDR region of the *rpoB* gene revealed that no mutations were detected in the *rpoB* gene of two isolates; even in MTBDR*plus* and TB-SPRINT. In the remaining three isolates, the mutations His526Asp, His526Asp and His526Tyr, and Asp516Ala were detected by sequencing. Likewise, the TB-SPRINT indicated the same mutation, His526Asp, for the first and second isolates; however, for the third one, there was an absence of WT probe hybridisation without mutation probe hybridisation in codon 516. In these three isolates, the MTBDR*plus* did not find the mutations in either the first or third isolates, classifying them both as drug susceptible; however, the same double mutation (His526Asp and His526Tyr) was found in the second isolate.

Similarly, in an evaluation of discordant results for isoniazid, the sequencing of *katG* and the *inhA* promoter region was performed in five resistant isolates by the DST. In four of them, the sequencing revealed no mutations, as was shown by the MTBDR*plus* and TB-SPRINT assays. In the one remaining isolate, the mutation *katG* Ser315Thr was detected by sequencing; the same mutation was found by TB-SPRINT, and no mutation was detected by MTBDR*plus*.

### Comparison of MTBDRplus and TB-SPRINT versus conventional DST

The results obtained upon comparing the performance of MTBDR*plus* and TB-SPRINT with those of conventional DST separately for each of the drugs and for MDR is presented in Table II.

**TABLE II:** Performance assay of MTBDR*plus* and tuberculosis-spoligo-rifampin-isoniazid typing (TB-SPRINT) molecular methods compared to phenotypical drug susceptibility testing (DST) results (n = 80)

	RIF resistance		INH resistance		MDR TB		
	TB-SPRINT	MTBDR*plus*	TB-SPRINT	MTBDR*plus*	TB-SPRINT	MTBDR*plus*	TB-SPRINT *versus* MTBDR*plus*
Sensitivity (%)	96.7	93.3	85.0	83.3	85.0	83.3	
	(92.1-101.2)	(87.0-99.6)	(76.0-94.0)	(73.9-92.8)	(76.0-94.0)	(73.9-92.8)	NA
Specificity (%)	100.0	100.0	100	100	100	100	
	(100.0-100.0)	(100.0-100.0)	(100-100)	(100-100)	(100-100)	(100-100)	NA
Accuracy (%)	97.5	95.0	88.8	87.5	88.8	87.5	
	(94.1-100.9)	(90.2-99.8)	(81.8-95.7)	(80.3-94.7)	(81.8-95.7)	(80.3-94.7)	NA
Kappa*a*	0.94	0.88	0.74	0.71	0.74	0.71	0.97
	(0.85-1.02)	(0.76-0.99)	(0.58-0.90)	(0.55-0.88)	(0.58-0.90)	(0.55-0.88)	(0.9-1.03)

a the criteria for kappa values were applied: kappa statistic (< 0,20 = poor; 0,21-0,40 = feeble; 0,41-0,60 = moderate; 0,61-0,80 = good; > 0,80-1.0 = very good);

*: values in parentheses are with 95% confidence intervals; INH: isoniazid; MDR TB: multidrug-resistant tuberculosis; NA: not applicable; RIF: rifampicin.

## DISCUSSION

This is the first study on the performance of resistance assessment by TB-SPRINT and MTBDR*plus* using the BACTEC^TM^ MGIT 960 System method as a reference on clinical *M. tuberculosis* isolates.

The results from MTBDR*plus* and TB-SPRINT confirm that the majority of mutations conferring RIF resistance in the present clinical isolate population (93% of the isolates) involve the 81 bp RRDR region (codons 507-533) of the *rpoB* gene, highlighting codon 531 as being more frequently affected (Zhang & Yew 2009, Gomgnimbou et al. 2013). Despite the higher complexity of the molecular basis of INH resistance, MTBDR*plus* could mostly identify the mutation and confirmed that major mutations associated with INH resistance were in codons of *katG* and *inhA* (Hillemann et al. 2005).

Results from MTBDR*plus* and TB-SPRINT are concordant with previous studies describing resistance mutation prevalence on MTC isolates from Brazil (Dalla Costa et al. 2009, Maschmann et al. 2013, de Freitas et al. 2014).

Concerning the sequencing results, the mutation Asp516Ala could not be identified either by MTBDR*plus* or TB-SPRINT because this less common mutation is not present in their mutation panel (Gomgnimbou et al. 2013, Aurin et al. 2014).

The comparison of the molecular techniques in the case of RIF resistance shows high sensitivity for both tests (Table II). In general, the results are very similar for both techniques, with slight differences essentially due to the difference in probe design (Table I). For RIF resistance, 42 mutations each were detected by MTBDR*plus* and 44 by TB-SPRINT, but the mutation panel is not 100% identical, as four and five mutations are targeted by MTBDR*plus* and TB-SPRINT, respectively. Our results are in agreement with the estimation that approximately 95% of RIF resistance is directly detected by known genetic mutations in the region of the gene *rpoB* (Cardoso et al. 2004). Earlier studies on TB-SPRINT and MTBDR*plus* also demonstrate the excellent ability of these two tests to detect the most prevalent mutations (Gomgnimbou et al. 2013, Aurin et al. 2014). In the case of the isolates that were MDR by phenotypic DST but WT by either MTBDR*plus* or TB-SPRINT, this could be due to the presence of mutations outside of the *rpoB* hotspot region to, another resistance mechanism such as efflux pumps, or even to the presence of heteroresistance (da Silva & Palomino 2011, Khan et al. 2013). The specificity of both techniques was excellent. The accuracy was similar for TB-SPRINT and MTBDR*plus* with Kappa, indicating a “very good” correlation between the results from molecular and phenotypic testing for RIF, as has been observed in other studies (Bwanga et al. 2009, Feuerriegel et al. 2012).

The performance of the MTBDR*plus* and TB-SPRINT in INH resistance exhibited a lower sensitivity for detection of RIF resistance in comparison with the conventional DST. The higher complexity of INH resistance mechanism and, therefore, mutations and/or other genes involved are also responsible for INH resistance (Zhang & Yew 2009) but not covered by the presently used methods, which could explain these discordant findings (Folkvardsen et al. 2013). Like RIF resistance, specificity for detection of INR-resistance by both methods was very high. Furthermore, TB-SPRINT and MTBDR*plus* showed good accuracy and correlation in comparison with the conventional DST. As stated before, additional mechanisms causing drug resistance have been described and include permeability changes of the cell envelope structure surrounding the bacillus, regulation of efflux pumps and the production of specific enzymes that metabolise otherwise toxic molecules (Louw et al. 2009, Nguyen & Pieters 2009, Srivastava et al. 2010, Müller et al. 2011). MDR-TB detection performances by TB-SPRINT and MTBDR*plus* were very similar to INH detection performances. This is due to INH detection being more difficult and thus acting as a limiting factor in MDR-TB detection. Although there were slightly discordant findings between the genotypic and phenotypic procedures, a good global correlation between the results from molecular and phenotypic testing for RIF has been observed in other studies (Bwanga et al. 2009, Feuerriegel et al. 2012). In addition, for the detection of INH resistance and MDR-TB, the TB-SPRINT showed slightly better performance than MTBDR*plus*, as corroborated by sequencing results of discordant isolates.

The good correlation between TB-SRINT and MTBDR*plus* also compared with conventional DST and demonstrates that these techniques are reliable for the detection of chromosomal mutations that confer drug resistance in MTC. Owing to high sensitivity for detection of rifampin resistance and high specificity for MDR of these molecular methods, it is expected that they could subserve the suitable treatment of MDR-TB patients. However, as there is still discordance between the conventional and molecular approaches of DST, molecular methods cannot presently replace phenotypic DST for diagnosis of MDR-TB. Instead, phenotypic and genotypic assays are complementary to accurately predict MDR-TB (Schön et al. 2017).

In conclusion, both methods exhibited a good correlation with the conventional DST. Moreover, TB-SPRINT and MTBDR*plus* are easily performed and are reliable for the simultaneous detection of RIF and INH mutations, even though these methods require technical expertise. Despite their good performance, neither TB-SPRINT nor MTBDRplus can replace the phenotypic DST. Instead, the molecular methods could be used in the guidance of therapy, which should be followed by a confirmation of phenotypic DST for all suspected MDR-TB patients, mainly in places with a high TB prevalence. Because of the size of Brazil and regional differences in the impact of the TB control programme, we suggest testing both methods and estimating the cost-effectiveness of MTBDR*plus* and TB-SPRINT in low and high MDR-TB prevalence areas of the country.
