# Erratum

**DOI:** 10.1590/0074-02760170062ER

**Published:** 2018-02-19

**Vol. 112(11): 769-774, 2017.**

**p. 769**

Phillip Noel Suffys

**should read:**

Philip Noel Suffys

